# 4-Methyl-*N*-[(2-oxo-1,3-thia­zolidin-3-yl)carbon­yl]benzene­sulfonamide

**DOI:** 10.1107/S1600536810014467

**Published:** 2010-04-24

**Authors:** Qing-Wu Chen, Jian-Quan Weng, Cheng-Xia Tan, De-Long Shen

**Affiliations:** a Zhejiang University of Technology, College of Chemical Engeering and Material Science, Hangzhou, Zhejiang 310014, People’s Republic of China

## Abstract

The asymmetric unit of the title compound, C_11_H_12_N_2_O_4_S_2_, contains two independent mol­ecules with similar dihedral angles of 76.7 (1) and 77.3 (1)° between the mean planes of the five- and six-membered rings. In both mol­ecules, the amino groups are involved in intra­molecular N—H⋯O hydrogen bonds. In the crystal structure, weak inter­molecular C—H⋯O hydrogen bonds link mol­ecules into ribbons extended along the *a* axis.

## Related literature

For a related structure, see: Gowda *et al.* (2010 ▶). For details of the synthesis, see: Chen & Shen (2008 ▶). For the biological activity of related compounds, see: Fujimoto & Shimizu (1978 ▶); Liu *et al.* (2007 ▶, 2009 ▶).
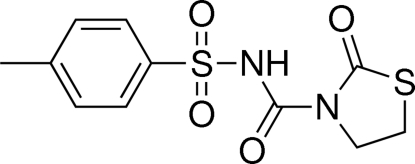


## Experimental

### 

#### Crystal data

C_11_H_12_N_2_O_4_S_2_
*M* *_r_* = 300.35
Triclinic, 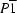

*a* = 9.5560 (4) Å
*b* = 9.6722 (4) Å
*c* = 14.6352 (6) Å
α = 88.7232 (10)°
β = 86.3267 (11)°
γ = 76.5399 (11)°
*V* = 1312.80 (9) Å^3^
*Z* = 4
Mo *K*α radiation
μ = 0.42 mm^−1^
*T* = 296 K
0.39 × 0.36 × 0.14 mm

#### Data collection

Rigaku R-AXIS RAPID diffractometer
Absorption correction: multi-scan (*ABSCOR*; Higashi, 1995 ▶) *T*_min_ = 0.825, *T*_max_ = 0.943
13020 measured reflections
5941 independent reflections
4475 reflections with *F* ^2^ > 2σ(*F* ^2^)
*R*_int_ = 0.019

#### Refinement

*R*[*F* ^2^ > 2σ(*F* ^2^)] = 0.048
*wR*(*F* ^2^) = 0.153
*S* = 1.00
5941 reflections
345 parameters
H-atom parameters constrained
Δρ_max_ = 0.52 e Å^−3^
Δρ_min_ = −0.38 e Å^−3^

### 

Data collection: *PROCESS-AUTO* (Rigaku, 2006 ▶); cell refinement: *PROCESS-AUTO*; data reduction: *CrystalStructure* (Rigaku, 2007 ▶); program(s) used to solve structure: *SHELXS97* (Sheldrick, 2008 ▶); program(s) used to refine structure: *SHELXL97* (Sheldrick, 2008 ▶); molecular graphics: *ORTEP-3* (Farrugia, 1997 ▶); software used to prepare material for publication: *WinGX* (Farrugia, 1999 ▶).

## Supplementary Material

Crystal structure: contains datablocks global, I. DOI: 10.1107/S1600536810014467/cv2707sup1.cif

Structure factors: contains datablocks I. DOI: 10.1107/S1600536810014467/cv2707Isup2.hkl

Additional supplementary materials:  crystallographic information; 3D view; checkCIF report

## Figures and Tables

**Table 1:** Hydrogen-bond geometry (Å, °)

*D*—H⋯*A*	*D*—H	H⋯*A*	*D*⋯*A*	*D*—H⋯*A*
N12—H12⋯O11	0.86	2.00	2.665 (3)	134
N32—H32⋯O31	0.86	1.91	2.608 (3)	137
C33—H332⋯O11	0.97	2.40	3.352 (3)	166
C11—H112⋯O33^i^	0.96	2.55	3.500 (4)	170
C32—H321⋯O12^ii^	0.97	2.60	3.384 (3)	138

Symmetry codes: (i) 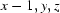; (ii) 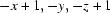.

**Fig. 1.**
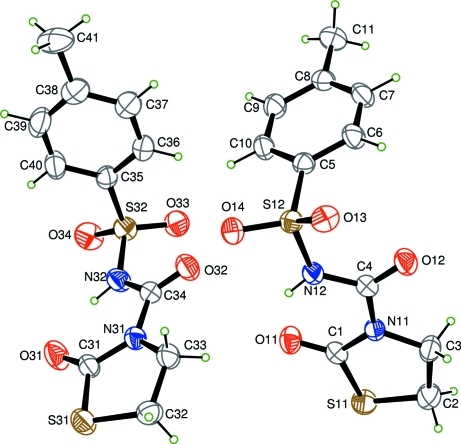
The molecular structure of (I), showing displacement ellipsoids drawn at the 50% probability level.

## supplementary crystallographic information

### Comment

2-Thiazolidione derivatives are known  as fungicides, insecticides and plant growth regulators (Chen *et al.* 2008; Fujimoto *et al.* 1978). Meanwhile, many pesticides contain amide fragments (Liu *et al.* 2007; Liu *et al.* 2009) . Herewith we present the title compound (I) - a new thiazolidione derivative  synthesized by the reaction of 2-thiazolidione and 4-methyl-benzenesulfonylisocyanate.

In (I) (Fig. 1), the carboxamide moiety is nearly coplanar with the thiazole ring [dihedral angle 7.2 (2)°]. The  C4-O12 bond length of 1.202 (3) Å is  normal for C=O double bond. The conformation of C—SO_2_—NH—C(O) fragment is similar to that observed in *N*-Benzoyl-2-chlorobenzenesulfonamide (Gowda *et al.*, 2010). Amino groups in two independent molecules are involved in intramolecular N—H···O hydrogen bonds (Table 1). In the crystal structure, weak intermolecular C—H···O hydrogen bonds (Table 1) link molecules into ribbons extended along the axis *a*.

### Experimental

The title compound was synthesized according to Chen *et al.* (2008). 2-Thiazolidiones (1.03 g, 0.01 mol) and 4-methyl- benzenesulfonylisocyanate (1.97 g,0.01 mol) were dissolved in anhydrous acetone(15 ml)with stirring. The mixture was then stirred at room temperature for 15 h. The solution was evaporated in a rotary evaporator at 30 degree under reduced pressure till 1.5 g of solide residue were obtained. This was washed three times with water and dried. Colourless single crystals suitable for crystallographic analysis were obtained by slow evaporation of an acetone-petroleum ether (1:20 v/v) solution.

### Refinement

All H atoms were geometrically positioned (C—H 0.93-0.97 Å; N—H 0.86 Å), and refined as riding, with U_iso_(H) = 1.2 U_eq_(C, N).

### Crystal data

**Table:**

C_11_H_12_N_2_O_4_S_2_	*Z* = 4
*M**_r_* = 300.35	*F*(000) = 624.00
Triclinic, *P*1	*D*_x_ = 1.519 Mg m^−^^3^
Hall symbol: -P 1	Mo *K*α radiation, λ = 0.71075 Å
*a* = 9.5560 (4) Å	Cell parameters from 10300 reflections
*b* = 9.6722 (4) Å	θ = 3.1–27.4°
*c* = 14.6352 (6) Å	µ = 0.42 mm^−^^1^
α = 88.7232 (10)°	*T* = 296 K
β = 86.3267 (11)°	Platelet, colourless
γ = 76.5399 (11)°	0.39 × 0.36 × 0.14 mm
*V* = 1312.80 (9) Å^3^	

### Data collection

**Table:**

Rigaku R-AXIS RAPID diffractometer	4475 reflections with *F*^2^ > 2σ(*F*^2^)
Detector resolution: 10.00 pixels mm^-1^	*R*_int_ = 0.019
ω scans	θ_max_ = 27.4°
Absorption correction: multi-scan (*ABSCOR*; Higashi, 1995)	*h* = −12→12
*T*_min_ = 0.825, *T*_max_ = 0.943	*k* = −10→12
13020 measured reflections	*l* = −18→18
5941 independent reflections	

### Refinement

**Table:**

Refinement on *F*^2^	*w* = 1/[σ^2^(*F*_o_^2^) + (0.0833*P*)^2^ + 0.8006*P*] where *P* = (*F*_o_^2^ + 2*F*_c_^2^)/3
*R*[*F*^2^ > 2σ(*F*^2^)] = 0.048	(Δ/σ)_max_ = 0.001
*wR*(*F*^2^) = 0.153	Δρ_max_ = 0.52 e Å^−^^3^
*S* = 1.00	Δρ_min_ = −0.38 e Å^−^^3^
5941 reflections	Extinction correction: *SHELXL97* (Sheldrick, 2008)
345 parameters	Extinction coefficient: 0.0029 (10)
H-atom parameters constrained	

### Special details

**Table:**

Geometry. ENTER SPECIAL DETAILS OF THE MOLECULAR GEOMETRY
Refinement. Refinement using reflections with *F*^2^ > 2.0 σ(*F*^2^). The weighted *R*-factor(wR), goodness of fit (*S*) and *R*-factor (gt) are based on *F*, with *F* set to zero for negative *F*. The threshold expression of *F*^2^ > 2.0 σ(*F*^2^) is used only for calculating *R*-factor (gt).

### Fractional atomic coordinates and isotropic or equivalent isotropic displacement parameters (Å^2^)

**Table:**

	*x*	*y*	*z*	*U*_iso_*/*U*_eq_	
S11	0.70694 (8)	0.42660 (8)	0.60372 (5)	0.0526 (2)	
S12	0.33746 (6)	0.09549 (6)	0.40275 (4)	0.03965 (16)	
S31	1.11065 (8)	−0.10590 (9)	0.27249 (5)	0.0583 (2)	
S32	0.72340 (8)	0.38204 (6)	0.06460 (5)	0.04988 (18)	
O11	0.6604 (2)	0.3008 (2)	0.45563 (12)	0.0524 (4)	
O12	0.2711 (2)	0.2641 (2)	0.57769 (12)	0.0538 (4)	
O13	0.2613 (2)	0.0224 (2)	0.46669 (12)	0.0489 (4)	
O14	0.4392 (2)	0.0178 (2)	0.33572 (12)	0.0505 (4)	
O31	1.0687 (2)	0.0667 (2)	0.13083 (14)	0.0704 (6)	
O32	0.6590 (2)	0.2350 (2)	0.24079 (13)	0.0598 (5)	
O33	0.6406 (2)	0.4885 (2)	0.12455 (14)	0.0596 (5)	
O34	0.8234 (2)	0.4156 (2)	−0.00386 (16)	0.0681 (6)	
N11	0.4892 (2)	0.3182 (2)	0.57686 (12)	0.0380 (4)	
N12	0.4356 (2)	0.1810 (2)	0.45973 (13)	0.0416 (4)	
N31	0.8833 (2)	0.0924 (2)	0.24434 (13)	0.0440 (4)	
N32	0.8270 (2)	0.2587 (2)	0.12745 (16)	0.0531 (5)	
C1	0.6163 (2)	0.3371 (2)	0.53329 (17)	0.0390 (5)	
C2	0.5766 (3)	0.4345 (4)	0.6988 (2)	0.0638 (8)	
C3	0.4517 (3)	0.3829 (3)	0.66798 (18)	0.0528 (6)	
C4	0.3885 (2)	0.2544 (2)	0.54018 (17)	0.0393 (5)	
C5	0.2141 (2)	0.2307 (2)	0.34841 (16)	0.0382 (5)	
C6	0.0829 (2)	0.2946 (3)	0.39243 (18)	0.0484 (6)	
C7	−0.0075 (2)	0.4065 (3)	0.3507 (2)	0.0514 (6)	
C8	0.0293 (2)	0.4558 (2)	0.26469 (18)	0.0430 (5)	
C9	0.1602 (2)	0.3878 (2)	0.22135 (18)	0.0492 (6)	
C10	0.2527 (2)	0.2758 (2)	0.26209 (17)	0.0462 (5)	
C11	−0.0668 (3)	0.5805 (3)	0.2195 (2)	0.0579 (7)	
C31	1.0186 (2)	0.0317 (2)	0.20360 (18)	0.0457 (5)	
C32	0.9758 (3)	−0.0719 (3)	0.36468 (19)	0.0549 (6)	
C33	0.8416 (3)	0.0248 (3)	0.32863 (18)	0.0552 (7)	
C34	0.7786 (2)	0.2013 (2)	0.20679 (17)	0.0431 (5)	
C35	0.6085 (2)	0.2939 (2)	0.01393 (17)	0.0454 (5)	
C36	0.4716 (3)	0.3001 (3)	0.05305 (19)	0.0519 (6)	
C37	0.3815 (3)	0.2319 (3)	0.0121 (2)	0.0613 (7)	
C38	0.4260 (3)	0.1557 (2)	−0.0680 (2)	0.0594 (7)	
C39	0.5622 (4)	0.1492 (3)	−0.1052 (2)	0.0691 (9)	
C40	0.6540 (3)	0.2172 (3)	−0.0651 (2)	0.0640 (8)	
C41	0.3275 (4)	0.0852 (3)	−0.1184 (3)	0.0843 (11)	
H6	0.0562	0.2622	0.4497	0.058*	
H7	−0.0950	0.4499	0.3806	0.062*	
H9	0.1861	0.4187	0.1634	0.059*	
H10	0.3397	0.2313	0.2320	0.055*	
H12	0.5215	0.1796	0.4378	0.050*	
H32	0.9161	0.2290	0.1092	0.064*	
H36	0.4407	0.3504	0.1069	0.062*	
H37	0.2893	0.2368	0.0385	0.074*	
H39	0.5935	0.0978	−0.1586	0.083*	
H40	0.7464	0.2113	−0.0913	0.077*	
H111	−0.0947	0.6578	0.2619	0.069*	
H112	−0.1513	0.5536	0.2011	0.069*	
H113	−0.0158	0.6098	0.1667	0.069*	
H201	0.6199	0.3745	0.7483	0.077*	
H202	0.5440	0.5317	0.7198	0.077*	
H301	0.4291	0.3126	0.7112	0.063*	
H302	0.3687	0.4622	0.6647	0.063*	
H321	0.9545	−0.1605	0.3867	0.066*	
H322	1.0094	−0.0257	0.4143	0.066*	
H331	0.7725	−0.0307	0.3161	0.066*	
H332	0.7986	0.0968	0.3737	0.066*	
H411	0.3605	−0.0162	−0.1140	0.101*	
H412	0.3288	0.1137	−0.1816	0.101*	
H413	0.2310	0.1135	−0.0915	0.101*	

### Atomic displacement parameters (Å^2^)

**Table:**

	*U*^11^	*U*^22^	*U*^33^	*U*^12^	*U*^13^	*U*^23^
S11	0.0476 (3)	0.0585 (4)	0.0574 (4)	−0.0234 (3)	−0.0027 (2)	−0.0058 (3)
S12	0.0403 (3)	0.0369 (2)	0.0419 (3)	−0.0088 (2)	−0.0039 (2)	−0.0024 (2)
S31	0.0489 (4)	0.0606 (4)	0.0561 (4)	0.0039 (3)	0.0030 (3)	0.0065 (3)
S32	0.0506 (3)	0.0487 (3)	0.0487 (3)	−0.0095 (2)	−0.0010 (2)	0.0084 (2)
O11	0.0514 (11)	0.0589 (11)	0.0493 (10)	−0.0207 (9)	0.0110 (8)	−0.0083 (8)
O12	0.0405 (10)	0.0667 (12)	0.0561 (11)	−0.0180 (9)	0.0075 (8)	−0.0143 (9)
O13	0.0557 (11)	0.0470 (9)	0.0482 (9)	−0.0205 (8)	−0.0062 (8)	0.0069 (8)
O14	0.0501 (10)	0.0454 (9)	0.0516 (10)	−0.0023 (8)	0.0004 (8)	−0.0113 (8)
O31	0.0567 (12)	0.0821 (15)	0.0581 (12)	0.0059 (11)	0.0174 (10)	0.0146 (11)
O32	0.0471 (11)	0.0673 (13)	0.0561 (11)	0.0019 (9)	0.0060 (9)	0.0031 (9)
O33	0.0660 (13)	0.0463 (10)	0.0644 (12)	−0.0075 (9)	−0.0076 (10)	−0.0037 (9)
O34	0.0636 (13)	0.0741 (14)	0.0673 (13)	−0.0216 (11)	0.0032 (10)	0.0216 (11)
N11	0.0359 (10)	0.0406 (10)	0.0373 (10)	−0.0087 (8)	0.0007 (7)	−0.0039 (8)
N12	0.0341 (10)	0.0474 (11)	0.0433 (10)	−0.0096 (8)	0.0000 (8)	−0.0083 (9)
N31	0.0410 (11)	0.0481 (11)	0.0408 (10)	−0.0073 (9)	0.0009 (8)	0.0011 (9)
N32	0.0419 (12)	0.0627 (14)	0.0506 (12)	−0.0056 (10)	0.0013 (9)	0.0092 (11)
C1	0.0360 (11)	0.0334 (11)	0.0471 (13)	−0.0078 (9)	−0.0001 (9)	0.0014 (10)
C2	0.0552 (17)	0.084 (2)	0.0548 (16)	−0.0200 (16)	−0.0016 (13)	−0.0195 (15)
C3	0.0529 (16)	0.0683 (17)	0.0407 (13)	−0.0223 (13)	0.0061 (11)	−0.0132 (12)
C4	0.0350 (11)	0.0391 (11)	0.0430 (12)	−0.0073 (9)	−0.0013 (9)	−0.0002 (10)
C5	0.0366 (11)	0.0393 (11)	0.0398 (11)	−0.0103 (9)	−0.0036 (9)	−0.0008 (9)
C6	0.0381 (13)	0.0599 (16)	0.0454 (13)	−0.0104 (11)	0.0060 (10)	0.0061 (12)
C7	0.0326 (12)	0.0616 (16)	0.0557 (15)	−0.0044 (11)	0.0051 (11)	0.0016 (13)
C8	0.0392 (12)	0.0450 (13)	0.0462 (13)	−0.0111 (10)	−0.0072 (10)	−0.0010 (10)
C9	0.0491 (14)	0.0543 (15)	0.0412 (13)	−0.0074 (12)	0.0018 (11)	0.0050 (11)
C10	0.0421 (13)	0.0502 (14)	0.0419 (12)	−0.0041 (11)	0.0065 (10)	−0.0002 (11)
C11	0.0495 (16)	0.0558 (16)	0.0660 (18)	−0.0064 (13)	−0.0095 (13)	0.0051 (14)
C31	0.0377 (12)	0.0492 (14)	0.0464 (13)	−0.0036 (10)	0.0021 (10)	−0.0010 (11)
C32	0.0491 (15)	0.0696 (18)	0.0451 (14)	−0.0122 (13)	−0.0052 (11)	0.0085 (13)
C33	0.0472 (15)	0.0683 (18)	0.0440 (14)	−0.0040 (13)	0.0049 (11)	0.0050 (13)
C34	0.0393 (13)	0.0457 (13)	0.0424 (12)	−0.0057 (10)	−0.0036 (10)	−0.0025 (10)
C35	0.0516 (14)	0.0437 (13)	0.0363 (12)	−0.0022 (11)	−0.0018 (10)	0.0036 (10)
C36	0.0560 (16)	0.0554 (15)	0.0430 (13)	−0.0113 (13)	0.0034 (11)	−0.0048 (11)
C37	0.0659 (19)	0.0620 (18)	0.0563 (17)	−0.0150 (15)	−0.0058 (14)	−0.0024 (14)
C38	0.076 (2)	0.0405 (14)	0.0592 (17)	−0.0052 (14)	−0.0201 (15)	0.0057 (12)
C39	0.088 (2)	0.0600 (18)	0.0517 (16)	−0.0011 (17)	−0.0036 (16)	−0.0183 (14)
C40	0.0616 (19)	0.0688 (19)	0.0528 (16)	0.0001 (15)	0.0099 (14)	−0.0099 (14)
C41	0.086 (2)	0.0507 (17)	0.121 (3)	−0.0136 (17)	−0.050 (2)	−0.0125 (19)

### Geometric parameters (Å, °)

**Table:**

S11—C1	1.750 (2)	C32—C33	1.518 (3)
S11—C2	1.797 (3)	C35—C36	1.383 (4)
S12—O13	1.424 (2)	C35—C40	1.379 (3)
S12—O14	1.4295 (18)	C36—C37	1.373 (5)
S12—N12	1.658 (2)	C37—C38	1.389 (4)
S12—C5	1.757 (2)	C38—C39	1.366 (5)
S31—C31	1.752 (2)	C38—C41	1.517 (5)
S31—C32	1.786 (2)	C39—C40	1.378 (5)
S32—O33	1.424 (2)	N12—H12	0.860
S32—O34	1.425 (2)	N32—H32	0.860
S32—N32	1.663 (2)	C2—H201	0.970
S32—C35	1.747 (3)	C2—H202	0.970
O11—C1	1.217 (3)	C3—H301	0.970
O12—C4	1.202 (3)	C3—H302	0.970
O31—C31	1.213 (3)	C6—H6	0.930
O32—C34	1.192 (3)	C7—H7	0.930
N11—C1	1.384 (3)	C9—H9	0.930
N11—C3	1.473 (3)	C10—H10	0.930
N11—C4	1.397 (3)	C11—H111	0.960
N12—C4	1.383 (3)	C11—H112	0.960
N31—C31	1.391 (3)	C11—H113	0.960
N31—C33	1.460 (3)	C32—H321	0.970
N31—C34	1.402 (3)	C32—H322	0.970
N32—C34	1.376 (3)	C33—H331	0.970
C2—C3	1.494 (4)	C33—H332	0.970
C5—C6	1.387 (3)	C36—H36	0.930
C5—C10	1.383 (3)	C37—H37	0.930
C6—C7	1.376 (3)	C39—H39	0.930
C7—C8	1.389 (3)	C40—H40	0.930
C8—C9	1.390 (3)	C41—H411	0.960
C8—C11	1.504 (3)	C41—H412	0.960
C9—C10	1.380 (3)	C41—H413	0.960
			
C1—S11—C2	93.82 (15)	C39—C38—C41	118.6 (2)
O13—S12—O14	120.18 (11)	C38—C39—C40	121.3 (2)
O13—S12—N12	108.77 (11)	C35—C40—C39	119.9 (3)
O13—S12—C5	109.42 (11)	S12—N12—H12	117.8
O14—S12—N12	103.25 (11)	C4—N12—H12	117.8
O14—S12—C5	109.48 (10)	S32—N32—H32	118.0
N12—S12—C5	104.49 (11)	C34—N32—H32	118.0
C31—S31—C32	93.95 (12)	S11—C2—H201	109.7
O33—S32—O34	120.96 (14)	S11—C2—H202	109.7
O33—S32—N32	108.22 (12)	C3—C2—H201	109.7
O33—S32—C35	109.34 (13)	C3—C2—H202	109.7
O34—S32—N32	102.86 (12)	H201—C2—H202	109.5
O34—S32—C35	109.16 (13)	N11—C3—H301	109.5
N32—S32—C35	105.02 (13)	N11—C3—H302	109.5
C1—N11—C3	116.0 (2)	C2—C3—H301	109.5
C1—N11—C4	126.5 (2)	C2—C3—H302	109.5
C3—N11—C4	117.2 (2)	H301—C3—H302	109.5
S12—N12—C4	124.46 (18)	C5—C6—H6	120.3
C31—N31—C33	116.3 (2)	C7—C6—H6	120.3
C31—N31—C34	125.9 (2)	C6—C7—H7	119.3
C33—N31—C34	117.4 (2)	C8—C7—H7	119.3
S32—N32—C34	123.93 (18)	C8—C9—H9	119.2
S11—C1—O11	123.1 (2)	C10—C9—H9	119.2
S11—C1—N11	111.07 (17)	C5—C10—H10	120.5
O11—C1—N11	125.8 (2)	C9—C10—H10	120.5
S11—C2—C3	108.6 (2)	C8—C11—H111	109.5
N11—C3—C2	109.4 (2)	C8—C11—H112	109.5
O12—C4—N11	121.0 (2)	C8—C11—H113	109.5
O12—C4—N12	124.1 (2)	H111—C11—H112	109.5
N11—C4—N12	114.9 (2)	H111—C11—H113	109.5
S12—C5—C6	120.75 (18)	H112—C11—H113	109.5
S12—C5—C10	118.64 (17)	S31—C32—H321	109.9
C6—C5—C10	120.6 (2)	S31—C32—H322	109.9
C5—C6—C7	119.5 (2)	C33—C32—H321	109.9
C6—C7—C8	121.3 (2)	C33—C32—H322	109.9
C7—C8—C9	118.0 (2)	H321—C32—H322	109.5
C7—C8—C11	121.7 (2)	N31—C33—H331	109.8
C9—C8—C11	120.3 (2)	N31—C33—H332	109.8
C8—C9—C10	121.7 (2)	C32—C33—H331	109.8
C5—C10—C9	119.0 (2)	C32—C33—H332	109.8
S31—C31—O31	123.45 (19)	H331—C33—H332	109.5
S31—C31—N31	110.43 (18)	C35—C36—H36	120.1
O31—C31—N31	126.1 (2)	C37—C36—H36	120.1
S31—C32—C33	107.76 (18)	C36—C37—H37	119.5
N31—C33—C32	108.3 (2)	C38—C37—H37	119.5
O32—C34—N31	121.5 (2)	C38—C39—H39	119.4
O32—C34—N32	125.0 (2)	C40—C39—H39	119.4
N31—C34—N32	113.5 (2)	C35—C40—H40	120.1
S32—C35—C36	120.2 (2)	C39—C40—H40	120.1
S32—C35—C40	120.2 (2)	C38—C41—H411	109.5
C36—C35—C40	119.6 (2)	C38—C41—H412	109.5
C35—C36—C37	119.8 (2)	C38—C41—H413	109.5
C36—C37—C38	120.9 (3)	H411—C41—H412	109.5
C37—C38—C39	118.5 (3)	H411—C41—H413	109.5
C37—C38—C41	122.8 (3)	H412—C41—H413	109.5
			
C1—S11—C2—C3	−7.7 (2)	S12—N12—C4—N11	179.73 (15)
C2—S11—C1—O11	−179.1 (2)	C31—N31—C33—C32	15.7 (3)
C2—S11—C1—N11	2.52 (19)	C33—N31—C31—S31	−4.9 (3)
O13—S12—N12—C4	−41.4 (2)	C33—N31—C31—O31	175.1 (3)
O13—S12—C5—C6	28.0 (2)	C31—N31—C34—O32	170.4 (2)
O13—S12—C5—C10	−154.4 (2)	C31—N31—C34—N32	−7.4 (4)
O14—S12—N12—C4	−170.14 (19)	C34—N31—C31—S31	−177.4 (2)
O14—S12—C5—C6	161.6 (2)	C34—N31—C31—O31	2.6 (4)
O14—S12—C5—C10	−20.7 (2)	C33—N31—C34—O32	−2.0 (4)
N12—S12—C5—C6	−88.3 (2)	C33—N31—C34—N32	−179.9 (2)
N12—S12—C5—C10	89.3 (2)	C34—N31—C33—C32	−171.2 (2)
C5—S12—N12—C4	75.4 (2)	S32—N32—C34—O32	−1.8 (4)
C31—S31—C32—C33	14.4 (2)	S32—N32—C34—N31	175.9 (2)
C32—S31—C31—O31	174.0 (2)	S11—C2—C3—N11	10.7 (3)
C32—S31—C31—N31	−6.0 (2)	S12—C5—C6—C7	175.8 (2)
O33—S32—N32—C34	45.7 (2)	S12—C5—C10—C9	−176.0 (2)
O33—S32—C35—C36	−19.4 (2)	C6—C5—C10—C9	1.6 (4)
O33—S32—C35—C40	160.9 (2)	C10—C5—C6—C7	−1.8 (4)
O34—S32—N32—C34	174.9 (2)	C5—C6—C7—C8	0.7 (4)
O34—S32—C35—C36	−153.8 (2)	C6—C7—C8—C9	0.6 (4)
O34—S32—C35—C40	26.5 (2)	C6—C7—C8—C11	−178.3 (2)
N32—S32—C35—C36	96.5 (2)	C7—C8—C9—C10	−0.8 (4)
N32—S32—C35—C40	−83.2 (2)	C11—C8—C9—C10	178.1 (2)
C35—S32—N32—C34	−71.0 (2)	C8—C9—C10—C5	−0.3 (4)
C1—N11—C3—C2	−9.6 (3)	S31—C32—C33—N31	−18.7 (3)
C3—N11—C1—S11	3.8 (2)	S32—C35—C36—C37	179.2 (2)
C3—N11—C1—O11	−174.5 (2)	S32—C35—C40—C39	−179.3 (2)
C1—N11—C4—O12	−167.6 (2)	C36—C35—C40—C39	1.0 (4)
C1—N11—C4—N12	12.8 (3)	C40—C35—C36—C37	−1.0 (4)
C4—N11—C1—S11	178.29 (17)	C35—C36—C37—C38	0.4 (4)
C4—N11—C1—O11	0.0 (3)	C36—C37—C38—C39	0.4 (4)
C3—N11—C4—O12	6.9 (3)	C36—C37—C38—C41	−176.7 (2)
C3—N11—C4—N12	−172.8 (2)	C37—C38—C39—C40	−0.5 (4)
C4—N11—C3—C2	175.3 (2)	C41—C38—C39—C40	176.7 (2)
S12—N12—C4—O12	0.1 (2)	C38—C39—C40—C35	−0.2 (4)

### Hydrogen-bond geometry (Å, °)

**Table:**

*D*—H···*A*	*D*—H	H···*A*	*D*···*A*	*D*—H···*A*
N12—H12···O11	0.86	2.00	2.665 (3)	134
N32—H32···O31	0.86	1.91	2.608 (3)	137
C33—H332···O11	0.97	2.40	3.352 (3)	166
C11—H112···O33^i^	0.96	2.55	3.500 (4)	170
C32—H321···O12^ii^	0.97	2.60	3.384 (3)	138

Symmetry codes: (i) *x*−1, *y*, *z*; (ii) −*x*+1, −*y*, −*z*+1.

## References

[bb1] Chen, Q. W. & Shen, D. L. (2008). *J. Zhejiang Univ. Technol.* **36**, 562–564.

[bb2] Farrugia, L. J. (1997). *J. Appl. Cryst.* **30**, 565.

[bb3] Farrugia, L. J. (1999). *J. Appl. Cryst.* **32**, 837–838.

[bb4] Fujimoto, E. & Shimizu, T. (1978). Jpn Patent JP 53127466.

[bb5] Gowda, B. T., Foro, S., Suchetan, P. A. & Fuess, H. (2010). *Acta Cryst.* E**66**, o794. 10.1107/S1600536810008731 PMC2984022 21580633

[bb6] Higashi, T. (1995). *ABSCOR* Rigaku Corporation, Tokyo, Japan.

[bb7] Liu, X. H., Chen, P. Q., Wang, B. L., Li, Y. H., Wang, S. H. & Li, Z. M. (2007). *Bioorg. Med. Chem. Lett.* **17**, 3784–3788. 10.1016/j.bmcl.2007.04.003 17512731

[bb8] Liu, X. H., Shi, Y. X., Ma, Y., He, G. R., Dong, W. L., Zhang, C. Y., Wang, B. L., Wang, S. H., Li, B. J. & Li, Z. M. (2009). *Chem. Biol. Drug Des.* **73**, 320–327. 10.1111/j.1747-0285.2009.00779.x 19207468

[bb9] Rigaku (2006). *PROCESS-AUTO* Rigaku Corporation, Tokyo, Japan.

[bb10] Rigaku (2007). *CrystalStructure* Rigaku Americas Corporation, The Woodlands, Texas, USA.

[bb11] Sheldrick, G. M. (2008). *Acta Cryst.* A**64**, 112–122. 10.1107/S0108767307043930 18156677

